# Investigation of singular ordinary differential equations by a neuroevolutionary approach

**DOI:** 10.1371/journal.pone.0235829

**Published:** 2020-07-09

**Authors:** Waseem Waseem, Muhammad Sulaiman, Poom Kumam, Muhammad Shoaib, Muhammad Asif Zahoor Raja, Saeed Islam

**Affiliations:** 1 Department of Mathematics, Abdul Wali Khan University Mardan, KP, Pakistan; 2 KMUTTFixed Point Research Laboratory, Department of Mathematics, Faculty of Science, King Mongkut’s University of Technology Thonburi (KMUTT), Bangkok, Thailand; 3 KMUTT-Fixed Point Theory and Applications Research Group, Theoretical and Computational Science Center (TaCS), Faculty of Science, King Mongkut’s University of Technology Thonburi (KMUTT), Bangkok, Thailand; 4 Department of Medical Research, China Medical University Hospital, China Medical University, Taichung, Taiwan; 5 Future Technology Research Center, National Yunlin University of Science and Technology, Yunlin, Taiwan, R.O.C.; 6 Department of Electrical and Computer Engineering, COMSATS University Islamabad, Attock, Pakistan; 7 Department of Mathematics, COMSATS University Islamabad, Attock, Pakistan; 8 Informetrics Research Group, Ton Duc Thang University, Ho Chi Minh City, Vietnam; 9 Faculty of Mathematics & Statistics, Ton Duc Thang University, Ho Chi Minh City, Vietnam; Universidad Veracruzana, MEXICO

## Abstract

In this research, we have investigated doubly singular ordinary differential equations and a real application problem of studying the temperature profile in a porous fin model. We have suggested a novel soft computing strategy for the training of unknown weights involved in the feed-forward artificial neural networks (ANNs). Our neuroevolutionary approach is used to suggest approximate solutions to a highly nonlinear doubly singular type of differential equations. We have considered a real application from thermodynamics, which analyses the temperature profile in porous fins. For this purpose, we have used the optimizer, namely, the fractional-order particle swarm optimization technique (FO-DPSO), to minimize errors in solutions through fitness functions. ANNs are used to design the approximate series of solutions to problems considered in this paper. We find the values of unknown weights such that the approximate solutions to these problems have a minimum residual error. For global search in the domain, we have initialized FO-DPSO with random solutions, and it collects best so far solutions in each generation/ iteration. In the second phase, we have fine-tuned our algorithm by initializing FO-DPSO with the collection of best so far solutions. It is graphically illustrated that this strategy is very efficient in terms of convergence and minimum mean squared error in its best solutions. We can use this strategy for the higher-order system of differential equations modeling different important real applications.

## 1 Introduction

Real-world problems which are modeled as a singular boundary value problem (BVP) of ordinary differential equations are often hard to solve. Such systems frequently arise in physics, or specifically astrophysics, thermodynamics, physical chemistry, nuclear technology, atomic energy, and all studies involving non-linear conic systems [1–4]. Singular BVPs are been tackled numerically and analytically by different researchers using techniques like the monotonic iterative method of Bessel functions [5], an improved iterative technique [6], homotopy perturbation method (HPM) [7], finite difference method with uniform mesh [8], monotonic iterative technique involving expansion of eigenfunction [9], modified adomian decomposition method (MADM) [10], Borel–Laplace transformation technique [11], and approximate power series solution method [12].

A review of all these numerical methods shows that they are deterministic and require prior information about the problem. Which is a disadvantage in case we do not have any information about a problem under consideration [13–16]. One example of such problem is the class of doubly non-linear singular differential equations. Meta-heuristic techniques are better alternatives for a variety of singular differential equations like doubly non-linear singular problems.

In this study, a soft computing approach based on hybridization of feed-forward neural networks and fractional-order darwinian PSO is suggested. To investigate the capability of our approach, we have solved three singular non-linear differential equation known as differential equations with doubly singularities. To further analyse our approach, we have solved nine subcases with different combinations of parameters. A real application is also considered in problem 4 to further highlight the effectiveness of our approach. We give a general representation of this system in Eq (1).

$$\begin{array}{c} {\left(p\left(x\right)y^{\prime }\left(x\right)\right)}^{\prime }=q\left(x\right)f\left(x,y\left(x\right)\right),\;\;\;\;\;0<x\leq 1, \end{array}$$ (1)

this differential equation is subject to the Dirichlet type of boundary conditions, which are given below.

$$\begin{array}{c} y\left(0\right)=a_{1},\;\;\;\;y\left(1\right)=c_{1}, \end{array}$$ (2)

some problems are also bounded by mixed boundary conditions as

$$\begin{array}{c} y(0)=0,\;\;\;\;ay(1)+by\prime (1)=c, \end{array}$$ (3)

here *a*, *a*_1_, *c*_1_ are non-zero positive real numbers and *b* ≥ 0. On the other hand, *c* can be any real number. When the value of *p*(0) is zero, the system becomes a singular differential equation. If *q*(*x*) is treated as a discontinuous function over the y-axis, then the problem stated in Eqs (1)–(3) becomes a doubly-singular type of differential equation.

From the above discussion, we have understood the singular doubly boundary value problem and based on this understanding we have developed our proposed soft computing approach to get better numerical solutions of these problems.

In the recent couple of years, alternate approaches based on artificial neural networks combined with heuristics and meta-heuristic are extensively developed to solve non-linear differential equations. Some important problems which are worth mentioning, include conduction problem in electrical engineering [17, 18], thermodynamics [19, 20], non-linear pantograph differential equations [21], models of atom known as Thomas-Fermi equations [22], Fuzzy logic based problems [23], Navier-stokes equations [23], Volterra differential equations [24, 25], problems in nanofluids [26], Fredholm integro-differential equations [27], non-linear Flierl-Petviashvili differential systems [28], problems in fractional control theory [29], bilinear programming differential systems [30], flow studies of non-linear differential system of Jeffery-Hamel problems [28], Bratu differential systems [31], differential systems in electromagnetism [32].

In [33], two techniques namely GA and SQP are combined to tackle the doubly non-linear singular differential equations. However, this combined algorithm takes more time and is computationally expensive. Also, these techniques are local search routines which stuck in a local minimum. By viewing all these contributions, it has led us to design an easy-to-use approach based on soft computing, which can produce better solutions with less computational time in solving these problems which are already handled by classical techniques. The main disadvantage of those classical techniques was their requirement of prior information about the problem in hand. In this paper, our proposed approach is used to solve a second-order non-linear differential equation with double singularities and complex boundary conditions.

Our approach aims to train the unknown weights in ANN by minimizing the error function through a well-balanced single meta-heuristic algorithm known as FO-DPSO [34, 35]. We have considered different case studies of non-linear doubly singular BVPs to check the capabilities of our approach. To examine the robustness of our approach, we have performed multiple simulations to get the best values of unknown weights. A real application is considered in problem 4 to further illustrate the effectiveness of our algorithm.

**Fig 1:**
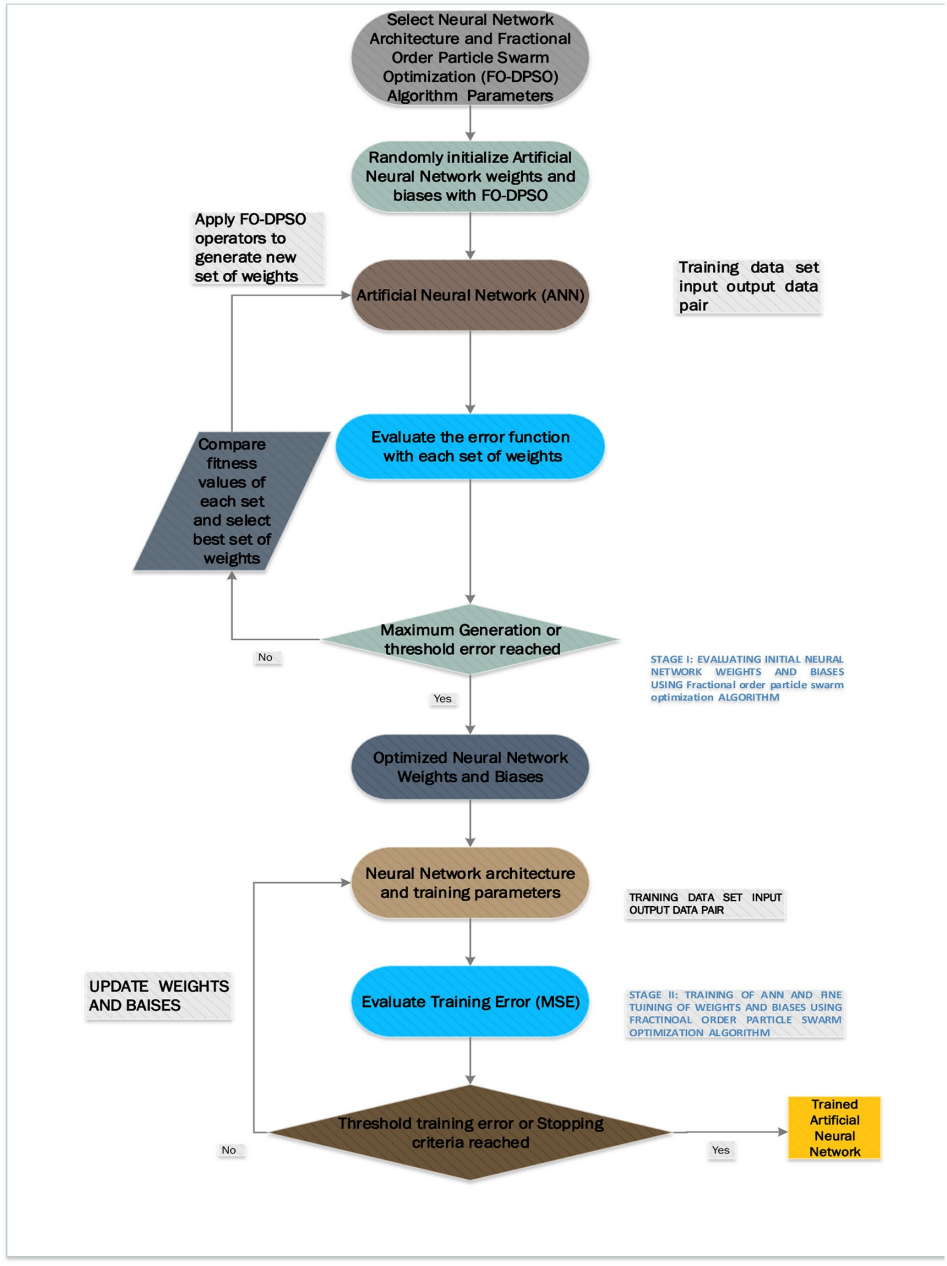
Graphical illustration of our soft computing procedure for doubly singular non-linear ODEs and Porous fin model.

**Fig 2:**
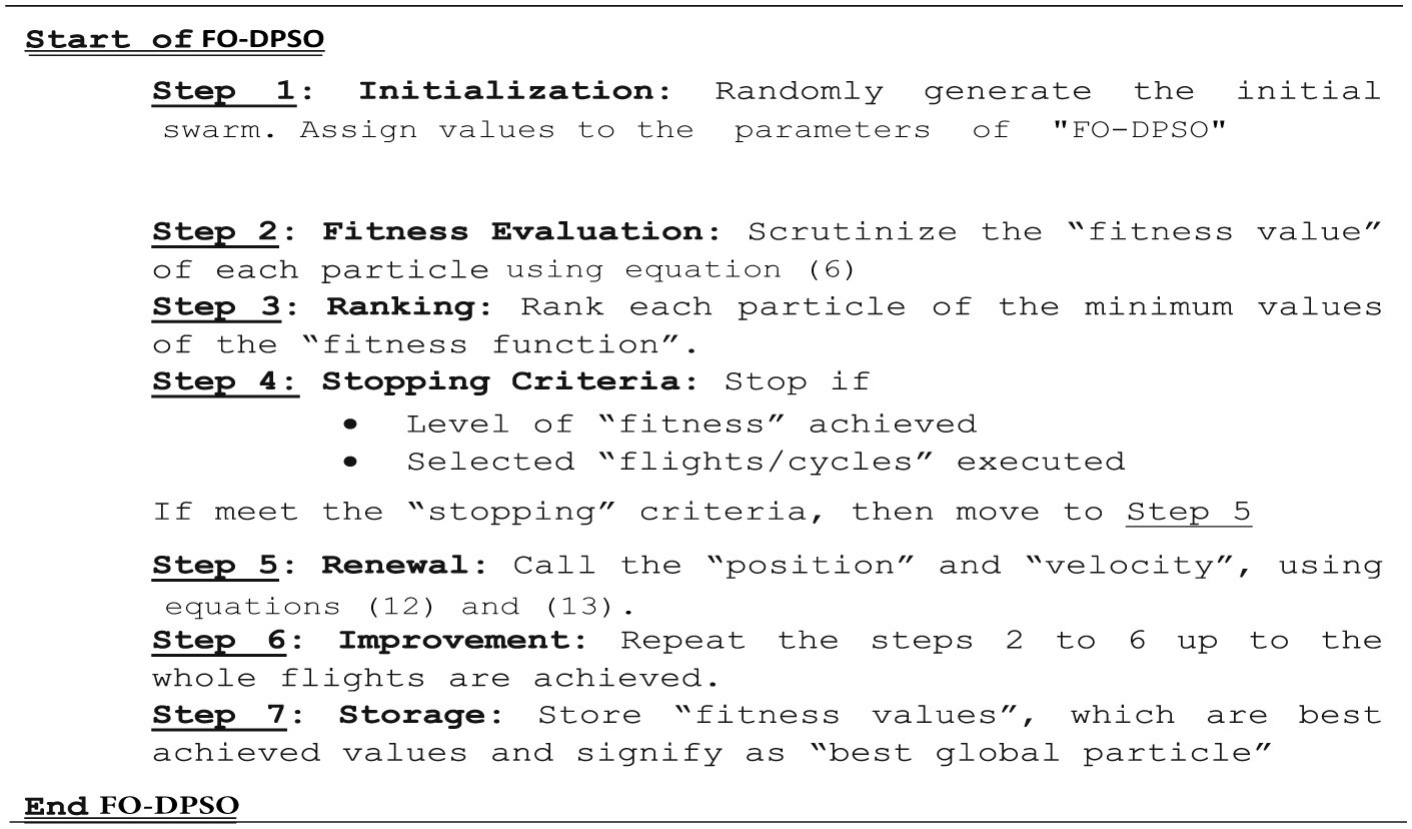
Pseudo-code of our soft computing technique.

**Fig 3:**
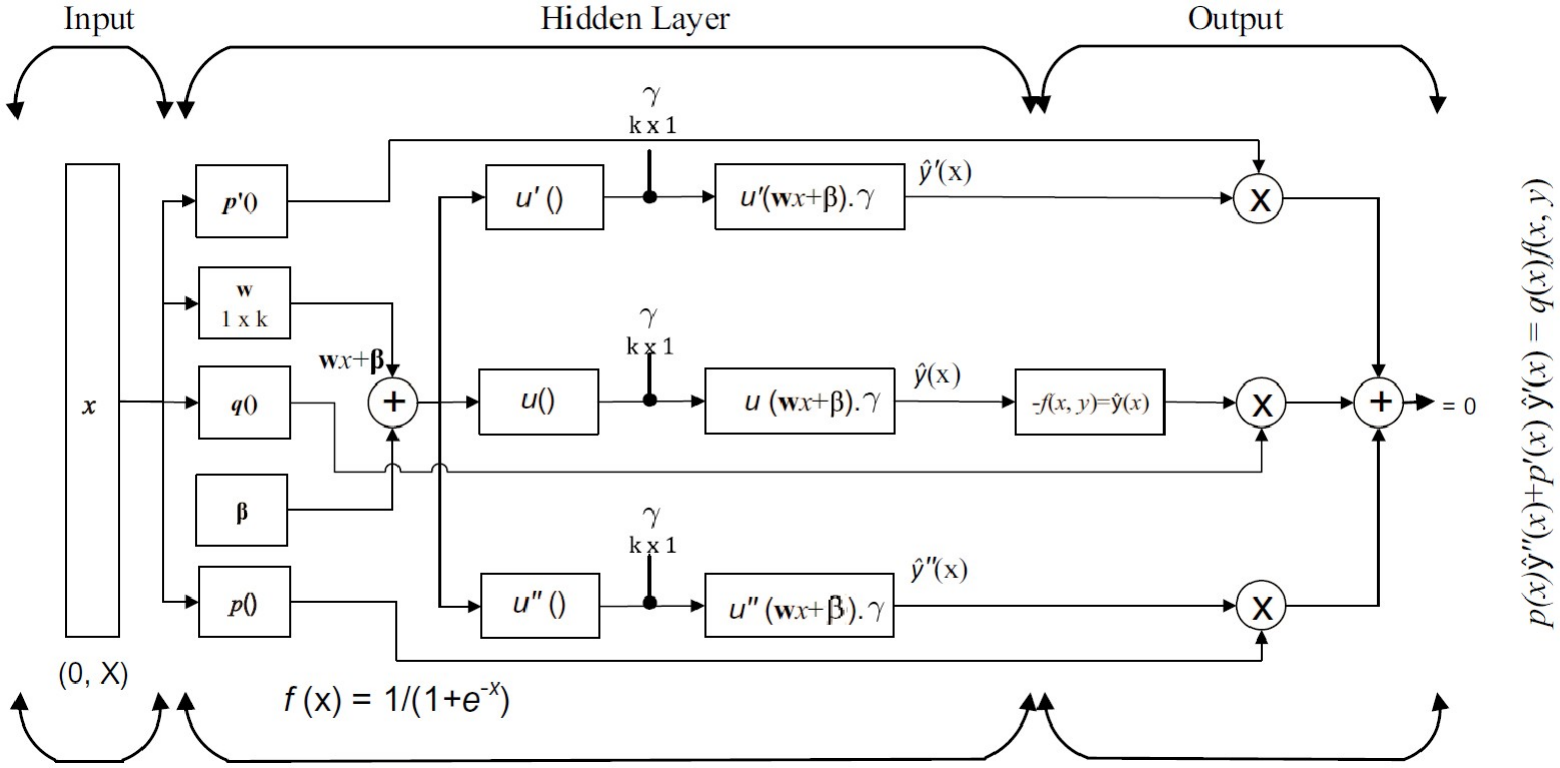
Neural network architecture.

Key contributions in this paper are given below:

- A theoretical and graphical model that explains why our soft computing approach works, and it is novel, is presented in section 2 (2.1, 2.2) and Figs (1), (2) and (3).
- Series solutions based on artificial neural networks are designed with the help of fractional-order particle swarm optimizer (FO-DPSO). Our novel soft computing approach is used to solve non-linear doubly singular differential equations, see Fig 1.
- A real application problem is considered to further elaborate on the competitiveness of our approach. In this problem, we have analyzed the temperature profile in a porous fin model, see Fig 8.
- We have compared our results with GA and a variant GA-SQP algorithm.
- The statistical analysis is presented in terms of absolute errors, global mean absolute error (*GMAE*), mean absolute error (MAE), and mean value of fitness (*M*_*fit*_).
- Computational times, maximum iterations took to solve our problems by ANN-based FO-DPSO are presented.
- Frequency plots of performance indicators fitted with normal distribution are graphically illustrated.

The rest of this paper is organized as, in section 2, mathematical modeling of approximate solution based on ANNs is illustrated. Fitness functions and FO-DPSO is briefly explained. Sections 3 and 4 contain problems description, numerical results for different case studies. Section 5, comprises the statistical analysis based on different performance indicators. Conclusions and future work are given in section 6.

## 2 The hybrid ANN and FO-DPSO approach

In this section, we have presented our novel approach. This approach constructs ANN-based approximate solutions with unknown weights for the doubly singular BVPs. The unknown weights are determined such that the approximate solution satisfies the problem with a less residual error. We have presented a detailed graphical abstract of the novel procedure in Fig 1.

### 2.1 ANN based approximation

Most of the real-world problems are mathematically modeled in the form of differential equations containing the derivative of integer and/ or fractional order. ANNs are frequently used to suggest approximate solutions for such problems [36–41].

In [42] the author presented a generalized method of neural networks to tackle both ODE’s as well as PDE’s. This method is based on approximation of a function and the capability of feedforward artificial neural network is to construct a solution of the differential equation which is differentiable and in a closed analytic form. Evolutionary optimization algorithm has been applied to train the weights and biases of the network, which results in minimum mean squared error and turn a good approximate solution of the problem.

The mathematical form of an approximate solution for BVPs with doubly singularities is suggested by feed-forward ANN as given in Eq (4),

$$\begin{array}{c} \hat{y}\left(x\right)=\sum_{i=1}^{k}\gamma_{i}u\left(w_{i}x+\beta_{i}\right), \end{array}$$ (4)

$$\begin{array}{c} \frac{d^{n}\hat{y}\left(x\right)}{dx^{n}}=\sum_{i=1}^{k}\gamma_{i}\frac{d^{n}}{dx^{n}}u\left(w_{i}x+\beta_{i}\right), \end{array}$$ (5)

*ŷ*(*x*) represents the approximate solution, *x* is the independent variable, *γ*_*i*_, *β*_*i*_ and *w*_*i*_ are the unknown weights, and Eq (5) expresses the *n*^*th*^ derivative of this approximate solution. A detailed discussion of neural networks is given in [36, 37, 41]. An activation function $u\left(x\right)=\frac{1}{1+e^{-}x}$ also known as Log-sigmoid mapping function is used to train the unknown weights. We substitute the approximate solutions in Eqs (1)–(3) to design an ANN-based solution for doubly singular BVPs. We show a detailed architecture of interaction among input-output and hidden stages of ANN in Fig 3. The objective function includes the minimization of mean squared errors in the approximate solutions.

$$\begin{array}{c} Minimize\;\;\;\;\;e=e_{1}+e_{2}, \end{array}$$ (6)

where *e*_1_, *e*_1_ are associated mean sqï»¿uared errors in ODE and boundary conditions, respectively.

$$e_{1}=\frac{1}{N}\sum_{m=1}^{N}((p_{m}\hat{y}\prime_{m})\prime -q_{m}f(x_{m},{\hat{y}}_{m}){)}^{2},$$ (7)

where *xϵ*(0, 1), step size *h* = 0.05, $N=\frac{1}{h}$, *p*_*m*_ = *p*(*x*_*m*_), *q*_*m*_ = *q*(*x*_*m*_), and *x*_*m*_ = *mh*.

Error due to boundary conditions is represented in Eq (8)

$$\begin{array}{c} e_{2}=\frac{1}{2}\left({\left({\hat{y}}_{0}-a_{1}\right)}^{2}+{\left({\hat{y}}_{N}-c_{1}\right)}^{2}\right). \end{array}$$ (8)

If the boundary conditions are mixed as discussed in Eq (3), then *e*_2_ can be approximated as,

$$e_{2}=\frac{1}{2}({\left(\hat{y}\prime_{0}\right)}^{2}+{\left(a{\hat{y}}_{N}+b\hat{y}\prime_{N}-c_{1}\right)}^{2}).$$ (9)

### 2.2 Search method of FO-DPSO

According to published findings, fractional calculus (FC) has received much interest to adapt it in the interpretation and solution of engineering challenges [43–45], applied mathematics, mechanical/dynamics [46, 47]. Grunwald-Letnikov defined a fractional derivative that contains fractional coefficients *αϵR*, a real number, by adjusting an unknown function *x*(*t*) as in Eq (10),

$$\begin{array}{c} D^{\alpha }\left[x\left(t\right)\right]=\lim_{h\to 0}[\frac{1}{h}\sum_{k=0}^{+\infty }\frac{{\left(-1\right)}^{k}\Gamma \left(\alpha +1\right)x\left(t-kh\right)}{\Gamma (k+1)\Gamma (\alpha -k+1)}], \end{array}$$ (10)

where the symbol Γ represent gamma function.

It is further elaborated that the series is characterized by bounded terms in Eq (10), if the derivative is of integer order. If *α* is fractional, infinite terms represent the result. It is therefore important to note that ordinary derivatives are operators which are local / instantaneous, whereas fractional operators represent a memory of past variations. With time, the memory of past instances declines. The Eq (11) determines a derivative for discrete instances. [48–52],

$$\begin{array}{c} D^{\alpha }\left[x\left(t\right)\right]=\frac{1}{T^{\alpha }}[\sum_{k=0}^{r}\frac{{\left(-1\right)}^{k}\Gamma \left(\alpha +1\right)x\left(t-kh\right)}{\Gamma (k+1)\Gamma (\alpha -k+1)}], \end{array}$$ (11)

The term *T* refers to the time intervals of events and *r* is number of truncated terms. Because of their memory retention properties, methods found in fractional calculus are useful in irretrievable and disorganised systems. Taking into account swarms ‘chaotic behavior in the Darwinian Particle swarms optimization algorithm, fractional calculus tools are appropriate to keep track of swarms’ past movements.

Taking into account the inertial weight in FO-DPSO *w* = 1, *T* as 1 and the research performed in [35, 53, 54], we have the following expression:

$$\begin{array}{c} D^{\alpha }\left[v_{t+1}^{n}\right]=\rho_{1}r_{1}\left({\overset{ˇ}{g}}_{t}^{n}-x_{t}^{n}\right)+\rho_{2}r_{2}\left({\overset{ˇ}{x}}_{t}^{n}-x_{t}^{n}\right)+\rho_{3}r_{3}\left({\overset{ˇ}{n}}_{t}^{n}-x_{t}^{n}\right). \end{array}$$ (12)

The empirical results of the algorithm are identical for *r* ≥ 4. The computational complexity also increases almost linearly, and therefore takes up the memory of *O*(*r*). Hence, it truncates the fifth term and onward for faster convergence. Thus *r*’s value is kept as 4. The inclusion of these four differential derivative terms means that the velocity term in FO-DPSO is as in Eq (13),

$$\begin{array}{cc} v_{t+1}^{n}= & \alpha v_{t}^{n}+\frac{1}{2}\alpha v_{t-1}^{n}+\frac{1}{6}\alpha \left(1-\alpha \right)v_{t-2}^{n}+\frac{1}{24}\alpha \left(1-\alpha \right)\left(2-\alpha \right)v_{t-3}^{n}+\rho_{1}r_{1}\left({\overset{ˇ}{g}}_{t}^{n}-x_{t}^{n}\right)\\ & +\rho_{2}r_{2}\left({\overset{ˇ}{x}}_{t}^{n}-x_{t}^{n}\right)+\rho_{3}r_{3}\left({\overset{ˇ}{n}}_{t}^{n}-x_{t}^{n}\right). \end{array}$$ (13)

## 3 Test problems and empirical results

In this section, we present three doubly singular type of differential equations, and their nine case studies are considered here to test the efficiency of our new approach.

### 3.1 Problem 1

This problem is a linear ODE with a doubly singularity with a polynomial forcing term. It is a boundary value problem with a non-homogenous ODE. Mathematically, it can be represented as in Eq (14) [2],

$$\{\begin{array}{l} y^{\prime \prime }\left(x\right)+\frac{1}{x}y^{\prime }\left(x\right)+\mu y=f\left(x\right),\\ \begin{array}{ll} y\left(0\right)=1, & y\left(1\right)=3, \end{array} \end{array}$$ (14)

where the exact solution is given in Eq (15)

$$\begin{array}{c} y\left(x\right)=x^{3}+x+1. \end{array}$$ (15)

Below we consider three cases of the problem by taking *μ* = −9, −1, 1 and forcing term as $f_{1}\left(x\right)=-9-9x^{3}+\frac{1}{x}$, $f_{2}\left(x\right)=-1-x^{3}+8x+\frac{1}{x}$ and $f_{3}\left(x\right)=1+x^{3}+10x+\frac{1}{x}$ respectively.

Case 1: From Eq (14) with *μ* = −9 and *f*(*x*) = *f*_1_(*x*) we get Eq (16),

$$\begin{array}{c} y^{\prime \prime }\left(x\right)+\frac{1}{x}y\prime \left(x\right)-9y=-9x^{3}-9+\frac{1}{x}. \end{array}$$ (16)

We give the error function which is used to measure the quality of the approximate solution in Eq (17)

$$\begin{array}{c} E=\frac{1}{N}\sum_{m=1}^{N}{\left(x_{m}{\hat{y}}_{m}^{\prime \prime }+{\hat{y}}_{m}^{\prime }-9x_{m}{\hat{y}}_{m}+9x_{m}+9x_{m}^{4}-1\right)}^{2}+\frac{1}{2}\left({\left({\hat{y}}_{0}-1\right)}^{2}+{\left({\hat{y}}_{N}-3\right)}^{2}\right) \end{array}$$ (17)

Case 2: From Eq (14) with *μ* = −1 and *f*(*x*) = *f*_2_(*x*) we get Eq (18),

$$\begin{array}{c} y^{\prime \prime }\left(x\right)+\frac{1}{x}y^{\prime }\left(x\right)-y=-x^{3}+8x-1+\frac{1}{x}. \end{array}$$ (18)

We give the error function which is used to measure the quality of the approximate solution in Eq (19)

$$\begin{array}{c} E=\frac{1}{N}\sum_{m=1}^{N}{\left(x_{m}{\hat{y}}_{m}^{\prime \prime }+{\hat{y}}_{m}^{\prime }-x_{m}{\hat{y}}_{m}+x_{m}+x_{m}^{4}-8x_{m}^{2}-1\right)}^{2}+\frac{1}{2}\left({\left({\hat{y}}_{0}-1\right)}^{2}+{\left({\hat{y}}_{N}-3\right)}^{2}\right) \end{array}$$ (19)

Case 3: From Eq (14) with *μ* = 1 and *f*(*x*) = *f*_3_(*x*) we get Eq (20),

$$\begin{array}{c} y^{\prime \prime }\left(x\right)+\frac{1}{x}y^{\prime }\left(x\right)+y=x^{3}+10x+1+\frac{1}{x}. \end{array}$$ (20)

We give the error function which is used to measure the quality of the approximate solution in Eq (21)

$$\begin{array}{c} E=\frac{1}{N}\sum_{m=1}^{N}{\left(x_{m}{\hat{y}}_{m}^{\prime \prime }+{\hat{y}}_{m}^{\prime }+x_{m}{\hat{y}}_{m}-x_{m}-x_{m}^{4}-10x_{m}^{2}-1\right)}^{2}+\frac{1}{2}\left({\left({\hat{y}}_{0}-1\right)}^{2}+{\left({\hat{y}}_{N}-3\right)}^{2}\right) \end{array}$$ (21)

**Fig 4:**
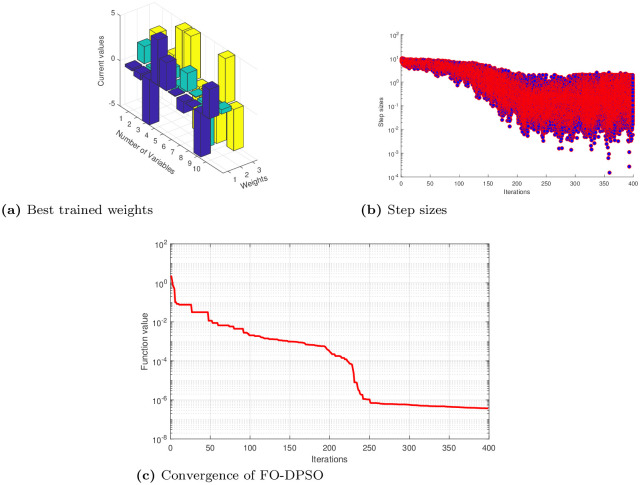
Best weights obtained, convergence of error values and step sizes used to reach the best solution for problem 1 case 1, 2, 3 using feed-forward ANNs based on FO-DPSO algorithm.

**Fig 5:**
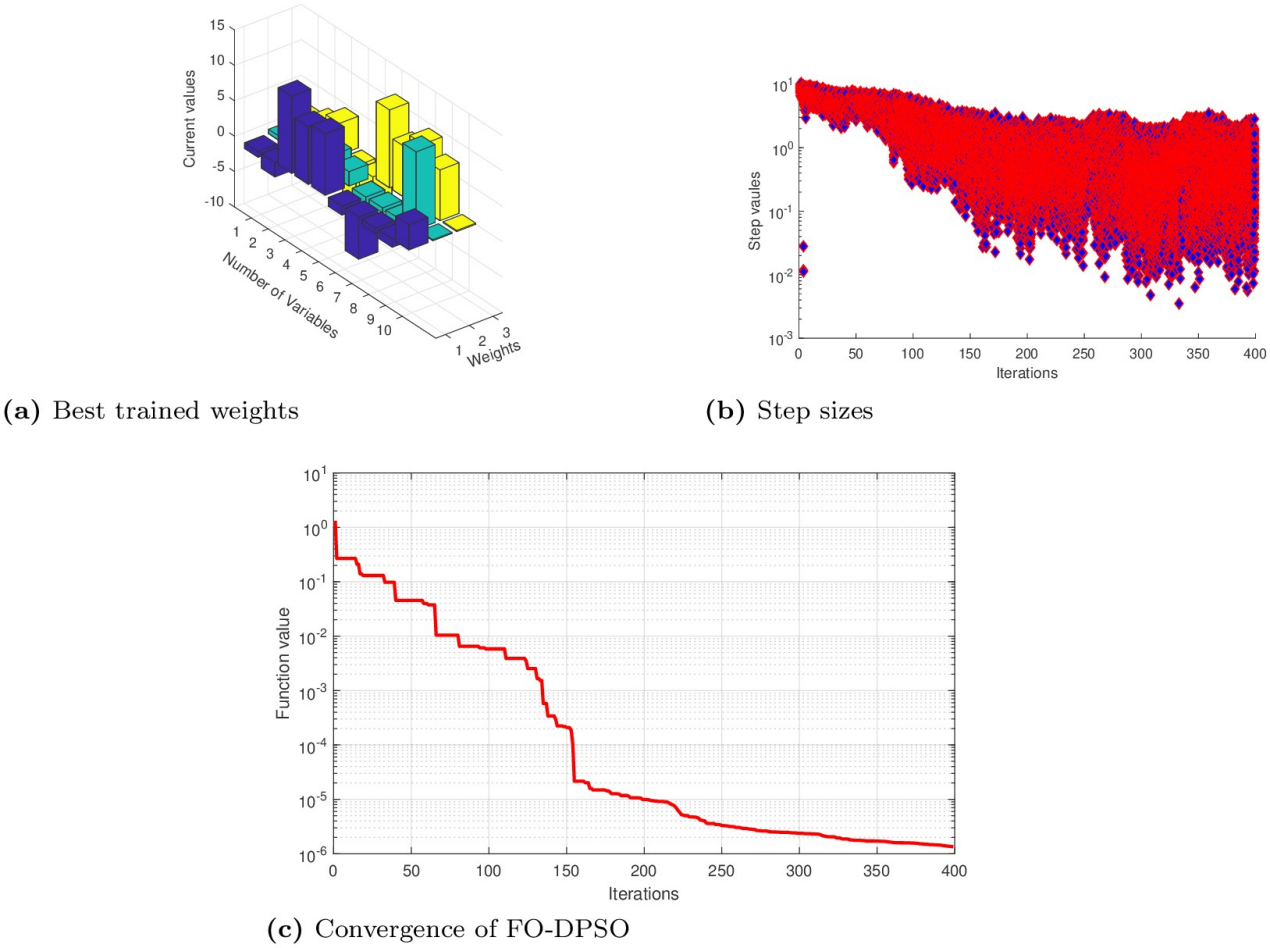
Best weights obtained, convergence of error values and step sizes used to reach the best solution for problem 2 case 1, 2, 3 using feed-forward ANNs based on FO-DPSO algorithm.

**Fig 6:**
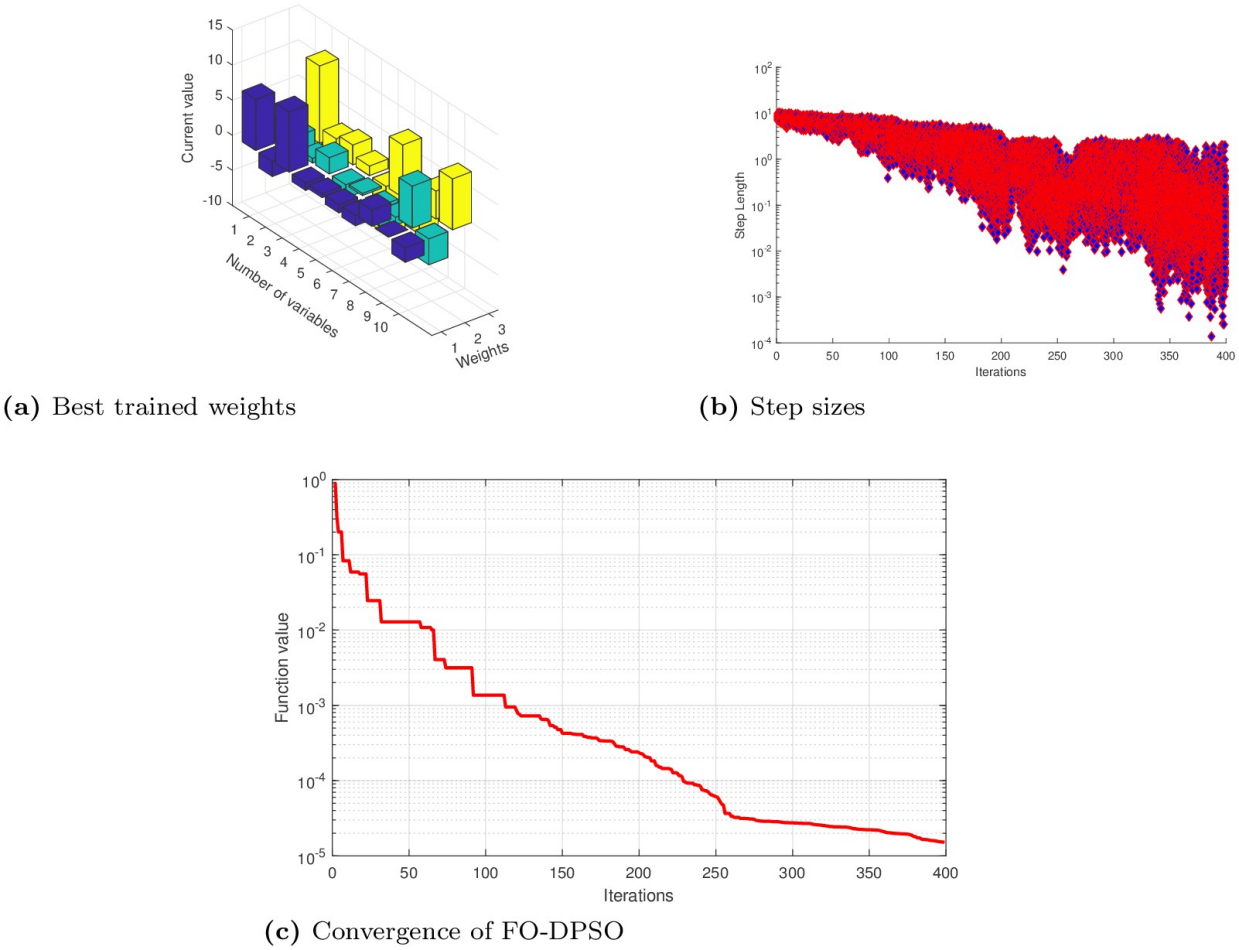
Best weights obtained, convergence of error values and step sizes used to reach the best solution for problem 3 case 1, 2, 3 using feed-forward ANNs based on FO-DPSO algorithm.

The unknown decision weights in error functions (17), (19) and (21) are determined by using the novel ANN based FO-DPSO approach. We briefly present approximate solutions for cases 1, 2, and 3 in Eqs (22), (23) and (24). We show the best values of weights along with convergence plots and step sizes in Figs 4, 5, 6 for Problems 1, 2, and 3. After getting the best weights, we used their values in approximate solutions given in Eq (4). For reproduction of our results, we have presented the complete solutions in the Appendix section without rounding off errors.

$$\begin{array}{c} \hat{y_{1}}\left(x\right)=\frac{-0.8218}{1+e^{-(0.6157x+1.57378)}}+\cdots +\frac{3.5514}{1+e^{-(0.1380x+0.1581)}} \end{array}$$ (22)

$$\begin{array}{c} \hat{y_{2}}\left(x\right)=\frac{5.597}{1+e^{-(2.3355x-3.0491)}}+\cdots +\frac{-0.3914}{1+e^{-(4.1093x+2.4261)}} \end{array}$$ (23)

$$\begin{array}{c} \hat{y_{3}}\left(x\right)=\frac{5.7670}{1+e^{-(1.9282x+4.0689)}}+\cdots +\frac{-2.8305}{1+e^{-(0.1369x+0.3719)}} \end{array}$$ (24)

Results got by FO-DPSO are compared with exact solutions, Genetic algorithm, its variant GA-SQP and are presented in Tables 1, 2, 3, 4, 5, 6, and 7 with step sizes h = 0.05 and 0.2, for problems 1, 2, 3 and 4. Input variable *x* is varied in interval [0 1]. The Absolute Error (AE) is calculated to highlight the better performance of our approach. Mathematically, it can be expressed as in Eq (25):

$$\begin{array}{c} AE=|y\left(x\right)-\hat{y}\left(x\right)|. \end{array}$$ (25)

**Fig 7:**
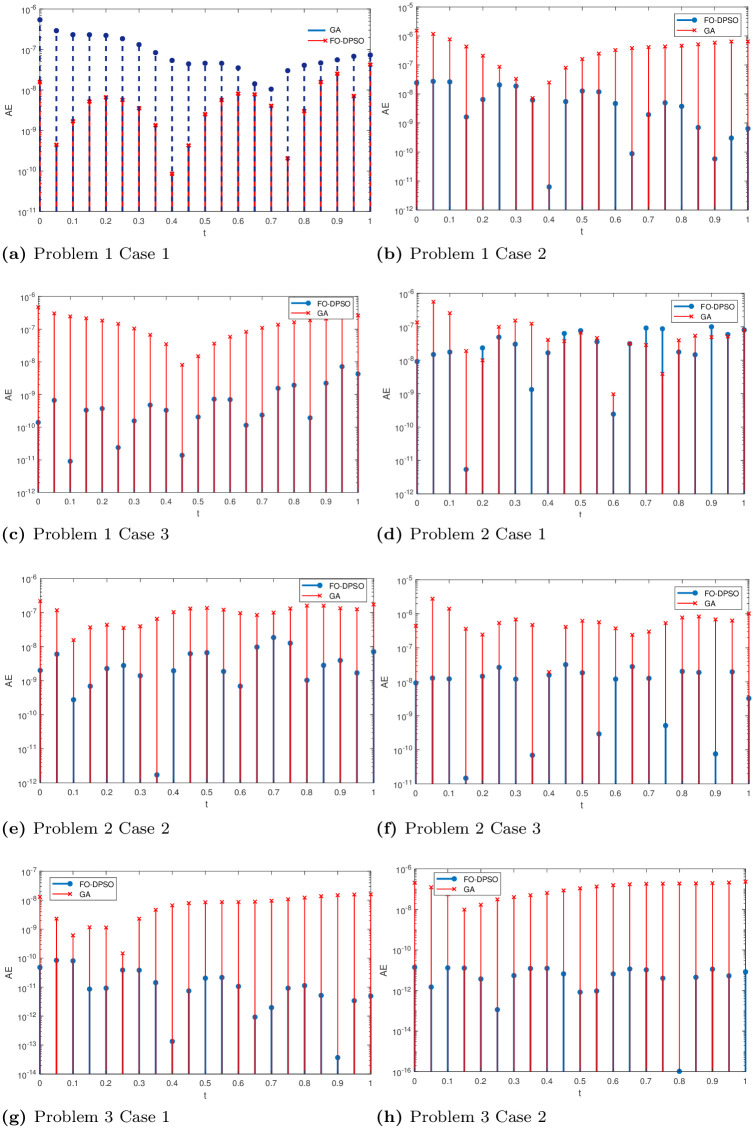
Graphical illustration of absolute errors in best solutions, for problem 1, 2 and 3 (Case 1, 2, 3), obtained by FO-DPSO and GA.

Values of AEs show better results in terms of accuracy of our approach. We give all values of AEs for step size h = 0.2 in Tables 2, 4 and 6. A graphical illustration of AEs with h = 0.05 is given in Fig 7.

**Table 1:** Empirical solutions for problem 1 (Case 1, 2, 3) achieved by FO-DPSO and GA. Which are compared with exact solutions for inputs *x* varying from 0 to 1 with a step size *h* = 0.05.

x		Case 1		Case 2		Case 3	
	Exact	FO-DPSO	GA	FO-DPSO	GA	FO-DPSO	GA
0	1	1.000031	1	1.000025	1	1	1
0.05	1.050125	1.050163	1.050124	1.050145	1.050125	1.050085	1.050122
0.1	1.101	1.101024	1.101	1.101008	1.101	1.100973	1.100999
0.15	1.153375	1.15338	1.153375	1.153373	1.153375	1.153365	1.153375
0.2	1.208	1.207991	1.208	1.207993	1.208	1.208002	1.208
0.25	1.265625	1.265613	1.265625	1.265617	1.265625	1.26563	1.265624
0.3	1.327	1.326993	1.327	1.326992	1.327	1.327002	1.326999
0.35	1.392875	1.392879	1.392875	1.392869	1.392875	1.39287	1.392875
0.4	1.464	1.464015	1.464	1.463995	1.464	1.463989	1.464
0.45	1.541125	1.541146	1.541125	1.54112	1.541125	1.541112	1.541125
0.5	1.625	1.625021	1.625	1.624993	1.625	1.62499	1.625001
0.55	1.716375	1.716391	1.716375	1.716365	1.716375	1.716369	1.716376
0.6	1.816	1.816008	1.816	1.815985	1.816	1.815998	1.816
0.65	1.924625	1.924625	1.924625	1.924607	1.924625	1.924626	1.924625
0.7	2.043	2.042996	2.043	2.042979	2.043	2.043	2.043
0.75	2.171875	2.171872	2.171875	2.171854	2.171875	2.171873	2.171876
0.8	2.312	2.312002	2.312	2.31198	2.312	2.311996	2.312001
0.85	2.464125	2.464133	2.464125	2.464105	2.464125	2.464121	2.464126
0.9	2.629	2.629013	2.629	2.62898	2.629	2.628997	2.629001
0.95	2.807375	2.807389	2.807375	2.807353	2.807375	2.807373	2.807376
1	3	3.000013	3	3	3	3	3.000001

**Table 2:** Absolute errors in results for problem 1 (Case 1, 2, 3) achieved by FO-DPSO and GA-SQP. Which are matched with exact solutions for inputs *x* varying from 0 to 1 with a step size *h* = 0.2.

x	Case 1 (AE)		Case 2 (AE)		Case 3 (AE)	
	FO-DPSO	GA-SQP	FO-DPSO	GA-SQP	FO-DPSO	GA-SQP
0	9.98E-09	1.36E-07	4.19E-08	2.17E-07	4.69E-09	4.40E-07
0.2	6.25E-07	9.96E-09	8.94E-08	4.39E-08	3.71E-07	2.45E-07
0.4	1.43E-06	4.07E-08	1.17E-06	1.03E-07	1.04E-06	1.94E-08
0.6	1.19E-06	9.64E-10	1.50E-06	9.63E-08	9.23E-07	3.75E-07
0.8	3.30E-07	3.95E-08	5.96E-07	1.60E-07	2.53E-07	7.77E-07
1	1.34E-08	8.07E-08	2.26E-08	1.73E-07	1.85E-08	1.02E-06

**Table 3:** Empirical solutions for problem 2 (Case 1, 2, 3) achieved by FO-DPSO and GA. Which are compared with exact solutions for inputs *x* varying from 0 to 1 with a step size *h* = 0.05.

x		Case 1		Case 2		Case 3	
	Exact	FO-DPSO	GA	FO-DPSO	GA	FO-DPSO	GA
0	1	1.000001	1	1.000002	1.000014	1	1.000001
0.05	1.051271	1.051271	1.051245	1.051272	1.05129	1.051271	1.051273
0.1	1.105171	1.105171	1.10516	1.105172	1.105187	1.105171	1.105172
0.15	1.161834	1.161834	1.161832	1.161835	1.161844	1.161834	1.161835
0.2	1.221403	1.221403	1.221406	1.221403	1.221408	1.221403	1.221403
0.25	1.284025	1.284026	1.284031	1.284026	1.284027	1.284025	1.284026
0.3	1.349859	1.349859	1.349866	1.349859	1.349859	1.349859	1.349859
0.35	1.419068	1.419068	1.419074	1.419068	1.419068	1.419067	1.419068
0.4	1.491825	1.491825	1.49183	1.491825	1.491825	1.491825	1.491825
0.45	1.568312	1.568312	1.568317	1.568312	1.568314	1.568312	1.568312
0.5	1.648721	1.648721	1.648725	1.648721	1.648723	1.648721	1.648721
0.55	1.733253	1.733253	1.733257	1.733253	1.733254	1.733253	1.733253
0.6	1.822119	1.822119	1.822123	1.822118	1.822119	1.822119	1.822118
0.65	1.915541	1.915541	1.915547	1.91554	1.91554	1.915541	1.91554
0.7	2.013753	2.013753	2.013761	2.013752	2.01375	2.013753	2.013752
0.75	2.117	2.117	2.117011	2.117	2.116997	2.117	2.117
0.8	2.225541	2.225541	2.225555	2.22554	2.225537	2.225541	2.22554
0.85	2.339647	2.339647	2.339663	2.339646	2.339642	2.339647	2.339646
0.9	2.459603	2.459603	2.459621	2.459603	2.459598	2.459603	2.459602
0.95	2.58571	2.58571	2.585728	2.585709	2.585704	2.58571	2.585708
1	2.718282	2.718282	2.7183	2.718281	2.718276	2.718282	2.718281

**Table 4:** Absolute errors in results for problem 2 (Case 1, 2, 3) achieved by FO-DPSO and GA. Which are matched with exact solutions for inputs *x* varying from 0 to 1 with a step size *h* = 0.2.

x	Case 1 (AE)		Case 2 (AE)		Case 3 (AE)	
	FO-DPSO	GA-SQP	FO-DPSO	GA-SQP	FO-DPSO	GA-SQP
0	6.45E-08	5.41E-07	2.48E-07	1.54E-06	8.66E-07	4.74E-07
0.2	9.19E-08	2.22E-07	2.67E-06	2.09E-07	3.23E-06	1.84E-07
0.4	1.05E-08	5.38E-08	8.41E-06	2.50E-08	1.88E-05	3.49E-08
0.6	2.22E-08	3.52E-08	5.33E-06	3.27E-07	2.65E-05	5.87E-08
0.8	5.91E-09	4.08E-08	9.07E-07	4.63E-07	1.17E-05	1.64E-07
1	2.77E-10	7.29E-08	2.94E-08	6.47E-07	1.05E-06	2.62E-07

**Table 5:** Empirical solutions for problem 3 (Case 1, 2, 3) achieved by FO-DPSO and GA. Which are compared with exact solutions for inputs *x* varying from 0 to 1 with a step size *h* = 0.05.

x		Case 1		Case 2		Case 3	
	Exact	FO-DPSO	GA	FO-DPSO	GA	FO-DPSO	GA
0	-1.38629	-1.386296	-1.38629	-1.386294	-1.38629	-1.386294	-1.38629
0.05	-1.39872	-1.398717	-1.39872	-1.398716	-1.39872	-1.398717	-1.39872
0.1	-1.41099	-1.410987	-1.41099	-1.410986	-1.41099	-1.410987	-1.41099
0.15	-1.42311	-1.423108	-1.42311	-1.423107	-1.42311	-1.423108	-1.42311
0.2	-1.43508	-1.435084	-1.43508	-1.435083	-1.43508	-1.435085	-1.43508
0.25	-1.44692	-1.446919	-1.44692	-1.446918	-1.44692	-1.446919	-1.44692
0.3	-1.45862	-1.458615	-1.45862	-1.458614	-1.45862	-1.458615	-1.45862
0.35	-1.47018	-1.470175	-1.47018	-1.470175	-1.47018	-1.470176	-1.47018
0.4	-1.4816	-1.481604	-1.4816	-1.481604	-1.4816	-1.481604	-1.4816
0.45	-1.4929	-1.492903	-1.4929	-1.492903	-1.4929	-1.492904	-1.4929
0.5	-1.50408	-1.504076	-1.50408	-1.504076	-1.50408	-1.504077	-1.50408
0.55	-1.51513	-1.515126	-1.51513	-1.515126	-1.51513	-1.515127	-1.51513
0.6	-1.52606	-1.526055	-1.52606	-1.526055	-1.52606	-1.526056	-1.52606
0.65	-1.53687	-1.536866	-1.53687	-1.536866	-1.53687	-1.536867	-1.53687
0.7	-1.54756	-1.547561	-1.54756	-1.547561	-1.54756	-1.547563	-1.54756
0.75	-1.55814	-1.558143	-1.55814	-1.558143	-1.55814	-1.558145	-1.55814
0.8	-1.56862	-1.568614	-1.56862	-1.568615	-1.56862	-1.568616	-1.56862
0.85	-1.57898	-1.578977	-1.57898	-1.578978	-1.57898	-1.578979	-1.57898
0.9	-1.58924	-1.589233	-1.58924	-1.589234	-1.58924	-1.589235	-1.58924
0.95	-1.59939	-1.599386	-1.59939	-1.599387	-1.59939	-1.599388	-1.59939
1	-1.60944	-1.609436	-1.60944	-1.609437	-1.60944	-1.609438	-1.60944

**Table 6:** Absolute errors in results for problem 3 (Case 1, 2, 3) achieved by FO-DPSO and GA-SQP. Which are matched with exact solutions for inputs *x* varying from 0 to 1 with a step size *h* = 0.2.

x	Case 1 (AE)		Case 2 (AE)		Case 3 (AE)	
	FO-DPSO	GA	FO-DPSO	GA	FO-DPSO	GA
0	1.39E-08	1.30E-08	7.68E-12	2.06E-07	8.55E-10	1.22E-09
0.2	6.01E-09	1.14E-09	5.51E-12	1.70E-08	5.98E-09	1.16E-09
0.4	2.79E-10	6.65E-09	4.90E-11	6.57E-08	6.20E-09	3.97E-09
0.6	1.16E-08	8.70E-09	3.55E-13	1.57E-07	2.43E-09	2.83E-09
0.8	1.44E-08	1.23E-08	7.72E-11	1.90E-07	9.90E-09	3.27E-09
1	1.65E-09	1.60E-08	1.97E-11	2.31E-07	1.49E-09	3.65E-09

**Table 7:** Comparison of solution obtained for the problem 4 of porous fin designed model using FO-DPSO.

*ξ*	Analytical	FO-DPSO	GA-SQP	GA
0	0.700465898	0.701211994	0.701171286	0.701382837
0.1	0.703355803	0.704109143	0.704069922	0.704273632
0.2	0.712042101	0.712810621	0.712779543	0.71301169
0.3	0.72657335	0.727352198	0.727330423	0.727587757
0.4	0.747026504	0.747792755	0.747775149	0.74800451
0.5	0.773500952	0.774210653	0.774192242	0.774320572
0.6	0.806110215	0.806702499	0.806683296	0.806665864
0.7	0.844971293	0.845383557	0.845368094	0.845227794
0.8	0.890191724	0.890388584	0.890380987	0.890213813
0.9	0.941854371	0.941871225	0.941870115	0.941798879
1	1	0.99999949	0.999999331	1.000067362
MSE		6.50E-08	1.00E-07	1.00E-04

FO-DPSO is better than GA-SQP and GA based approach as our approach is more accurate in solving problem 1. Results of AE show that ANN based FO-DPSO has produced values laying in ranges 10^−7^ *to* 10^−12^, 10^−8^ *to* 10^−12^ and 10^−8^ *to* 10^−11^ for problem 1, case 1, 2, and 3 respectively. We establish that ANN based on FO-DPSO is a successful technique for solving the problem under consideration.

### 3.2 Problem 2

In this problem, we consider a doubly singular ODE with boundary values and variable coefficients. It is a homogenous differential equation of second order. Mathematically, this problem can be represented as in Eq (26) [2],

$$\{\begin{array}{l} {\left(x^{\mu }y^{\prime }\left(x\right)\right)}^{\prime }=\nu x^{\mu +\nu -2}\left(\nu x^{\nu }+\mu +\nu -1\right)y\;0<x\leq 1\;\mu ,\nu >0\\ y\left(0\right)=1,\;y\left(1\right)=e. \end{array}$$ (26)

An exact solution for this problem is suggested in [2], Eq (27)

$$\begin{array}{c} y=e^{x^{\nu }}. \end{array}$$ (27)

We formulate three cases according to the variable coefficients *μ*, *ν*. We have tested the proposed method by solving these three cases.

Case 1: choosing *μ* = 0.5 and *ν* = 1 in problem (26), we get,

$$\begin{array}{c} \sqrt{x}y^{\prime \prime }\left(x\right)+\frac{1}{2\sqrt{x}}y^{\prime }\left(x\right)=\frac{1}{\sqrt{x}}\frac{2x+1}{2}y. \end{array}$$ (28)

The error function to judge the quality of the solutions is formulated as

$$\begin{array}{c} E=\frac{1}{N}\sum_{m=1}^{N}{\left(2x_{m}{\hat{y}}_{m}^{\prime \prime }+{\hat{y}}_{m}^{\prime }-2x_{m}{\hat{y}}_{m}-{\hat{y}}_{m}\right)}^{2}+\frac{1}{2}\left({\left({\hat{y}}_{0}-1\right)}^{2}+{\left({\hat{y}}_{N}-e\right)}^{2}\right). \end{array}$$ (29)

Case 2: choosing *μ* = 0.75 and *ν* = 1 in problem (26), we get,

$$\begin{array}{c} x^{3/4}y^{\prime \prime }\left(x\right)+\frac{3}{4x^{1/4}}y^{\prime }\left(x\right)=\frac{1}{x^{1/4}}\left(x+\frac{3}{4}\right)y. \end{array}$$ (30)

The error function to judge the quality of the solutions is formulated as

$$\begin{array}{c} E=\frac{1}{N}\sum_{m=1}^{N}{\left(4x_{m}{\hat{y}}_{m}^{\prime \prime }+3{\hat{y}}_{m}^{\prime }-4x_{m}{\hat{y}}_{m}-3{\hat{y}}_{m}\right)}^{2}+\frac{1}{2}\left({\left({\hat{y}}_{0}-1\right)}^{2}+{\left({\hat{y}}_{N}-e\right)}^{2}\right). \end{array}$$ (31)

Case 3: choosing *μ* = 0.25 and *ν* = 1 in problem (26), we get,

$$\begin{array}{c} x^{1/4}y^{\prime \prime }\left(x\right)+\frac{1}{4x^{3/4}}y^{\prime }\left(x\right)=\frac{1}{x^{3/4}}\left(x+\frac{1}{4}\right)y. \end{array}$$ (32)

The error function to judge the quality of the solutions is formulated as

$$\begin{array}{c} E=\frac{1}{N}\sum_{m=1}^{N}{\left(4x_{m}{\hat{y}}_{m}^{\prime \prime }+{\hat{y}}_{m}^{\prime }-4x_{m}{\hat{y}}_{m}-{\hat{y}}_{m}\right)}^{2}+\frac{1}{2}\left({\left({\hat{y}}_{0}-1\right)}^{2}+{\left({\hat{y}}_{N}-e\right)}^{2}\right). \end{array}$$ (33)

The unknown decision weights in error functions (29), (31) and (33) are determined by using the novel ANN based FO-DPSO approach. Values of AE show better results in terms of accuracy of the corresponding approach. We give all values of solutions and AEs (for h = 0.2) in Tables 3 and 4 for problem 2. FO-DPSO is better than GA-SQP and GA based approach by solving problem 2 more accurately. Results of AE show that ANN-based FO-DPSO has produced, ranges in 10^−8^ *to* 10^−11^, 10^−8^ *to* 10^−11^ and 10^−9^ *to* 10^−11^ respectively. We establish it that ANN-based on FO-DPSO is a successful technique for solving the problem under consideration.

### 3.3 Problem 3

In this problem, we consider a nonlinear doubly singular ODE with boundary values and variable coefficients. It is a homogenous differential equation of second order. Mathematically, this problem can be represented as in Eq (34) [2],

$$\{\begin{array}{l} {\left(x^{\mu }y^{\prime }\left(x\right)\right)}^{\prime }=\nu x^{\mu +\nu -2}e^{y}\left(e^{y}x^{\nu }-\mu -\nu +1\right)\;0<x\leq 1\;\mu ,\nu >0\\ y\left(0\right)=ln\left(\frac{\nu }{4}\right),\;y\left(1\right)=ln\left(\frac{\nu }{5}\right). \end{array}$$ (34)

An exact solution for this problem is suggested in [2], Eq (35)

$$\begin{array}{c} y=ln\frac{\nu }{4+x^{\nu }}. \end{array}$$ (35)

We formulate three cases according to the variable coefficients *μ*, *ν*. We have tested the proposed method by solving these three cases,

Case 1: choosing *μ* = 0.25 and *ν* = 1 in problem (34), we get,

$$\{\begin{array}{l} x^{1/4}y^{\prime \prime }\left(x\right)+\frac{1}{4x^{3/4}}y^{\prime }\left(x\right)=\frac{e^{y}}{x^{3/4}}\left(e^{y}x-\frac{1}{4}\right)\;0<x\leq 1\;\mu ,\nu >0\\ y\left(0\right)=ln\left(\frac{1}{4}\right),\;y\left(1\right)=ln\left(\frac{1}{5}\right). \end{array}$$ (36)

The error function to judge the quality of the solutions is formulated as

$$\begin{array}{c} E=\frac{1}{N}\sum_{m=1}^{N}{\left(4x_{m}{\hat{y}}_{m}^{\prime \prime }+{\hat{y}}_{m}^{\prime }-4x_{m}e^{{\hat{y}}_{m}}+e^{{\hat{y}}_{m}}\right)}^{2}+\frac{1}{2}\left({\left({\hat{y}}_{0}-ln\frac{1}{4}\right)}^{2}+{\left({\hat{y}}_{N}-ln\frac{1}{5}\right)}^{2}\right). \end{array}$$ (37)

Case 2: choosing *μ* = 0.5 and *ν* = 1 in problem (34), we get,

$$\{\begin{array}{l} x^{1/2}y^{\prime \prime }\left(x\right)+\frac{1}{2x^{1/2}}y^{\prime }\left(x\right)=\frac{e^{y}}{x^{1/2}}\left(e^{y}x-\frac{1}{2}\right)\;0<x\leq 1\;\mu ,\nu >0\\ y\left(0\right)=ln\left(\frac{1}{4}\right),\;y\left(1\right)=ln\left(\frac{1}{5}\right). \end{array}$$ (38)

The error function to judge the quality of the solutions is formulated as

$$\begin{array}{c} E=\frac{1}{N}\sum_{m=1}^{N}{\left(2x_{m}{\hat{y}}_{m}^{\prime \prime }+{\hat{y}}_{m}^{\prime }-2x_{m}e^{{\hat{y}}_{m}}+e^{{\hat{y}}_{m}}\right)}^{2}+\frac{1}{2}\left({\left({\hat{y}}_{0}-ln\frac{1}{4}\right)}^{2}+{\left({\hat{y}}_{N}-ln\frac{1}{5}\right)}^{2}\right). \end{array}$$ (39)

Case 3: choosing *μ* = 0.75 and *ν* = 1 in problem (34), we get,

$$\{\begin{array}{l} x^{3/4}y^{\prime \prime }\left(x\right)+\frac{1}{2x^{1/4}}y^{\prime }\left(x\right)=\frac{e^{y}}{x^{1/4}}\left(e^{y}x-\frac{3}{4}\right)\;0<x\leq 1\;\mu ,\nu >0\\ y\left(0\right)=ln\left(\frac{1}{4}\right),\;y\left(1\right)=ln\left(\frac{1}{5}\right). \end{array}$$ (40)

The error function to judge the quality of the solutions is formulated as

$$\begin{array}{c} E=\frac{1}{N}\sum_{m=1}^{N}{\left(4x_{m}{\hat{y}}_{m}^{\prime \prime }+3{\hat{y}}_{m}^{\prime }-4x_{m}e^{{\hat{y}}_{m}}+3e^{{\hat{y}}_{m}}\right)}^{2}+\frac{1}{2}\left({\left({\hat{y}}_{0}-ln\frac{1}{4}\right)}^{2}+{\left({\hat{y}}_{N}-ln\frac{1}{5}\right)}^{2}\right). \end{array}$$ (41)

$$\begin{array}{c} \hat{y}\left(x\right)=\frac{-1.7690}{1+e^{-(-1.6186x-4.3759)}}+\frac{0.6479}{1+e^{-(-0.3783x-0.1091)}} \end{array}$$ (42)

$$\begin{array}{c} \hat{y}\left(x\right)=\frac{-2.8314}{1+e^{-(0.8127x+2.7334)}}+\frac{0.1693}{1+e^{-(1.0845x+0.3734)}} \end{array}$$ (43)

$$\begin{array}{c} \hat{y}\left(x\right)=\frac{-0.2739}{1+e^{-(1.9006x+2.8037)}}+\frac{3.2499}{1+e^{-(0.4887x-4.5624)}} \end{array}$$ (44)

The unknown decision weights in error functions (37), (39) and (41) are determined by using the novel ANN based FO-DPSO approach. We briefly present approximate solutions for all cases in Eqs (42), (43) and (44). Values of AEs show better performance in terms of accuracy of our approach. We give all solutions and AEs (for h = 0.2) in Tables 5, and 6 for problem 3. FO-DPSO is better than GA and its variant GA-SQP by solving problem 3 more accurately. Results of AE show that ANN-based FO-DPSO has produced values laying in ranges 10^−11^ *to* 10^−13^, 10^−11^ *to* 10^−16^ and 10^−10^ *to* 10^−15^ respectively. We establish it that ANN-based on FO-DPSO is a successful technique for solving the problem under consideration.

## 4 Mathematical model of the porous fin

**Fig 8:**
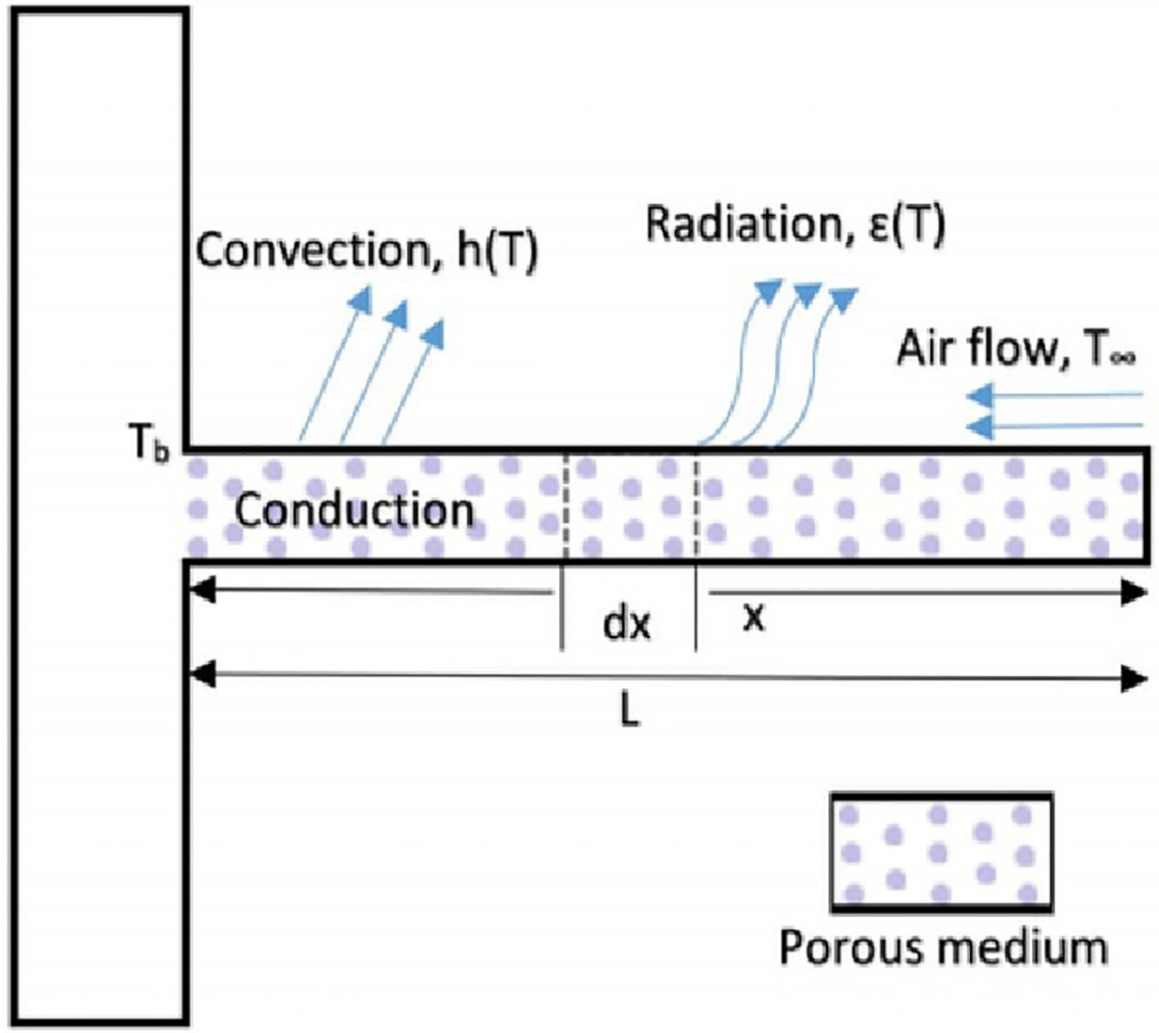
Design of a porous fin.

**Problem 4**: The schematic diagram of straight fin problem possessing the arbitrary cross-sectional area *A*_*c*_, perimeter *P*, and length *b*, is presented in Fig 8, [55]. The fin is joined with the base surface having the temperature *T*_*b*_, and extends into fluid having temperature *T*_*a*_, and its tip is insulated. The energy balance equation is written as:

$$\begin{array}{c} A_{c}\frac{d}{dx}[k\left(T\right)\frac{dT}{dx}]-Ph(T-T_{a})=0. \end{array}$$ (45)

In the Eq (45), *k*(*T*) indicates the temperature-dependent thermal conductivity and h represents the coefficient of heat transfer. It is considered that the thermal conductivity for the fin material is expressed:

$$\begin{array}{c} k\left(T\right)=k_{b}[1+\lambda (T-T_{b})]. \end{array}$$ (46)

In the expression (46), *k*_*b*_ represents the thermal conductivity at the ambient fluid temperature of the fin, and λ is standing for the variation of the thermal conductivity. Using the non-dimensional variables:

$$\begin{array}{c} \theta =\frac{T-T_{a}}{T_{b}-T_{a}},\;\xi =\frac{x}{b},\;\mu =\lambda (T_{b}-T_{a}),\;\;\text{and}\;\;\psi =(\frac{Phb^{2}}{k_{a}A_{c}}{)}^{1/2}, \end{array}$$ (47)

consequently the Eq (45) reduces into the following form:

$$\begin{array}{c} \frac{d^{2}\theta }{d\xi^{2}}+\mu \theta \frac{d^{2}\theta }{d\xi^{2}}+\mu (\frac{d\theta }{d\xi }{)}^{2}-\psi^{2}\theta ,\;0\leq \xi \leq 1, \end{array}$$ (48)

with the boundary conditions

$$\begin{array}{c} \frac{d\theta }{d\xi }{|}_{\xi -0}=0\;\;\text{and}\;\;\theta {|}_{\xi -1}=1. \end{array}$$ (49)

**Fig 9:**
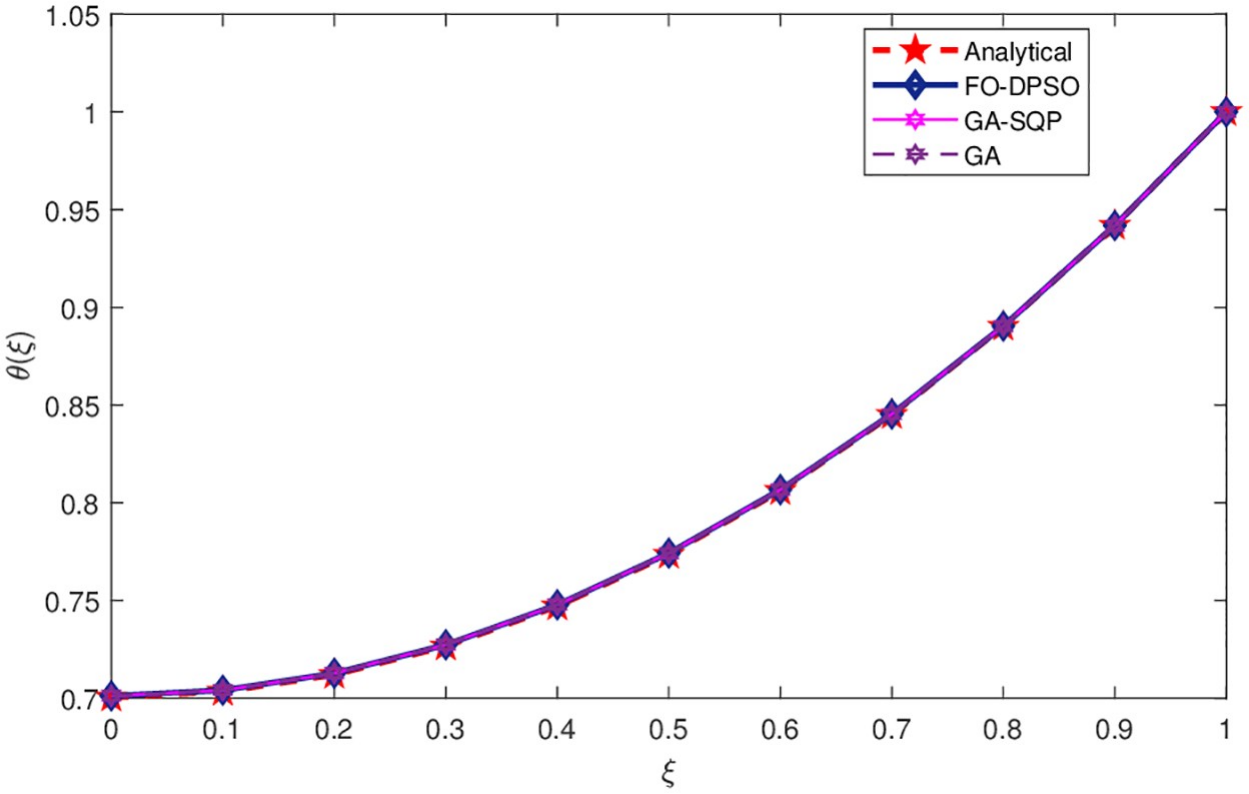
Solution obtained by our proposed method and other comparative algorithms for porous fin model.

We have solved a porous fin model using ANNs based FO-DPSO approach. The unknown weights are tuned by the FO-DPSO algorithm. Results obtained by FO-DPSO are compared with GA-SQP and GA and are given in Table 7. The graphical illustration of this model is given in Fig 8. Solutions obtained for this problem are plotted in Fig 9. Among the three algorithms, FO-DPSO performed well and gave us minimum error as compared to the other algorithms, see Fig 9.

## 5 Statistical analysis

**Fig 10:**
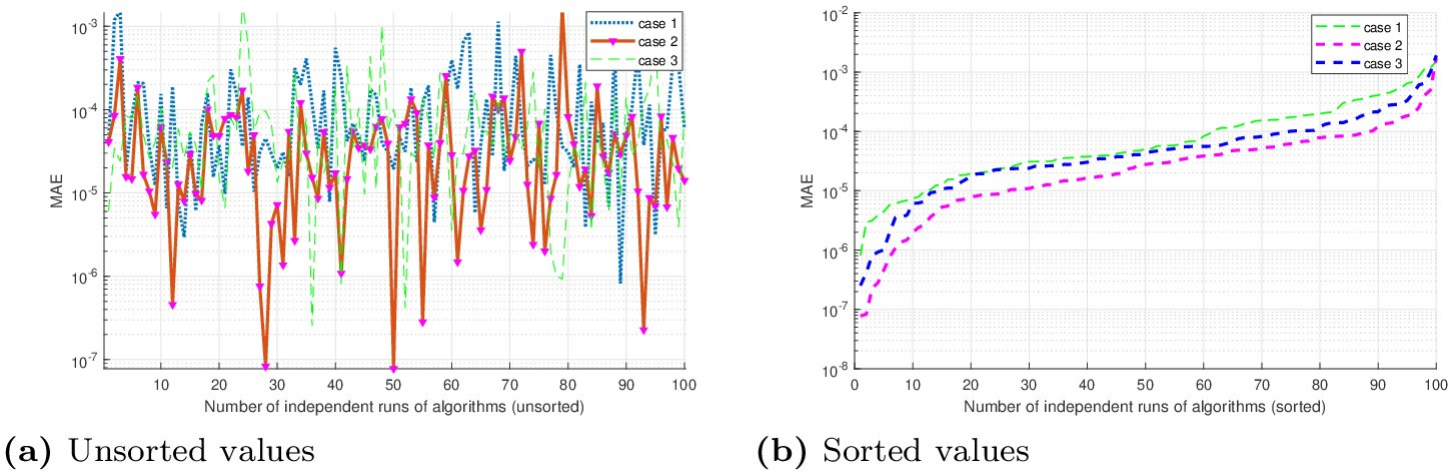
Graphical illustration of sorted absolute errors in solutions, for problem 1 (Case 1, 2, 3), obtained by FO-DPSO during 100 runs.

**Fig 11:**
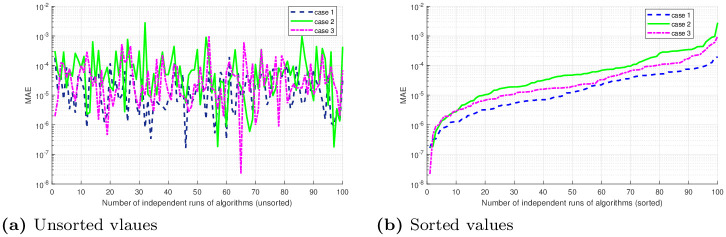
Graphical illustration of sorted absolute errors in solutions, for problem 2 (Case 1, 2, 3), obtained by FO-DPSO during 100 runs.

**Fig 12:**
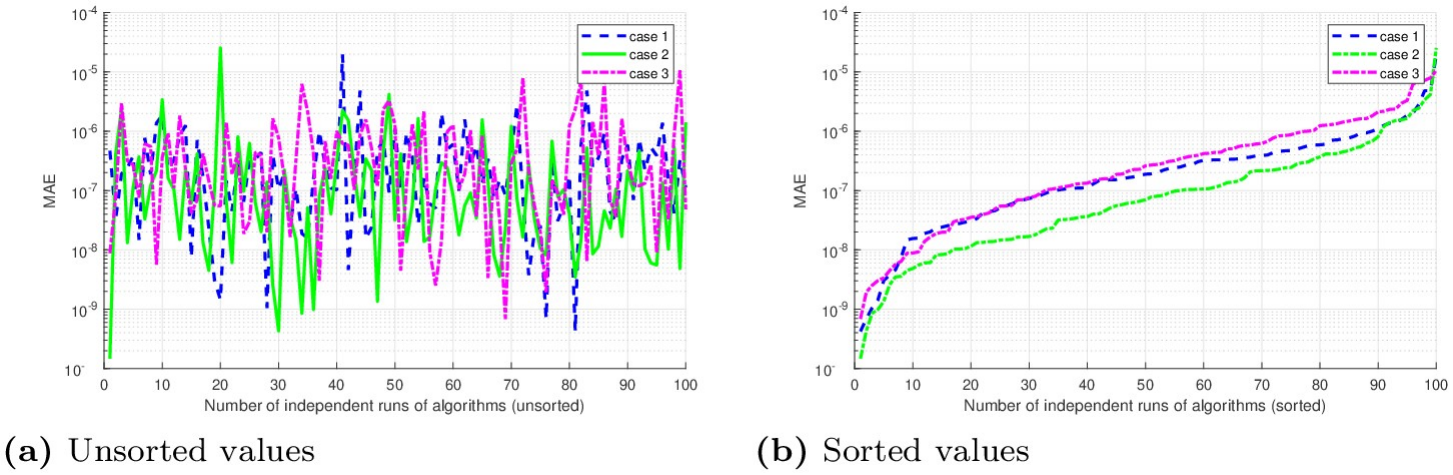
Graphical illustration of sorted absolute errors in solutions, for problem 3 (Case 1, 2, 3), obtained by FO-DPSO during 100 runs.

Performance indicators like global mean absolute error (GMAE), mean absolute error (MAE), and mean value of fitness (*M*_*fit*_) are used to asses the performance of ANNs based on FO-DPSO approach. We use these indicators on the data we have got through 100 independent simulations to determine the stability and robustness of our approach. We present MAE values in terms of sorted and unsorted form, see Figs 10, 11 and 12. Hence, the sorted results are presented in Figs 10b, 11b and 12b, while the unsorted errors in the solutions are given in Figs 10a, 11a and 12a, respectively. To further elaborate the difference between the errors obtained, and those reported in the literature, we have used the log scale plots for MAEs. From our graphical analysis, we get the minimum values of MAEs and better fitness for all problems. The performance of our approach is statistically analyzed in terms of the best minimum value, mean, and standard deviation (SD). This further validates our claim that our approach is better in convergence rate and has produced accurate results for all three BVPs with doubly singularities, and porous fin model. We present statistical results in terms of GMAE, Mean-time, Max iterations in Tables 8, 9 and 10. Our experimental outcome dictates that the ANNs based FO-DPSO approach has consistently produced better solutions to the non-linear ODEs and a real application problem.

**Table 8:** Performance indicators based on proposed results for Problem 1.

Type Parameters	Case 1	Case 2	Case 3
GMAE Values	9.40E-06	3.0273E-06	6.6613E-06
STD	5.6686E-06	2.7162E-06	5.3095E-06
Mfit VALUES	4.0931E-08	4.2931E-09	1.2824E-08
STD	3.2671E-08	4.6459E-09	9.6663E-09
Mean Time	27.8	30	28.4
Max Iteration	1000	1000	1000

**Table 9:** Performance indicators based on proposed results for Problem 2.

Type Parameters	Case 1	Case 2	Case 3
GMAE Values	1.51E-06	4.164E-06	3.207E-06
STD	9.542E-07	3.328E-06	2.047E-06
Mfit VALUES	7.747E-09	8.493E-09	1.037E-09
STD	1.005E-08	9.361E-09	1.729E-09
Mean Time	40.9	27.7	30.7
Max Iteration	1000	1000	1000

**Table 10:** Performance indicators based on proposed results for Problem 3.

Type Parameters	Case 1	Case 2	Case 3
GMAE Values	1.48E-08	5.4029E-09	1.2547E-08
STD	1.101E-08	3.7189E-09	1.0439E-08
Mfit VALUES	1.9996E-11	6.9589E-12	3.2591E-11
STD	2.4734E-11	4.8198E-12	6.6736E-11
Mean Time	29.5	29.9	28.9
Max Iteration	1000	1000	1000

**Fig 13:**
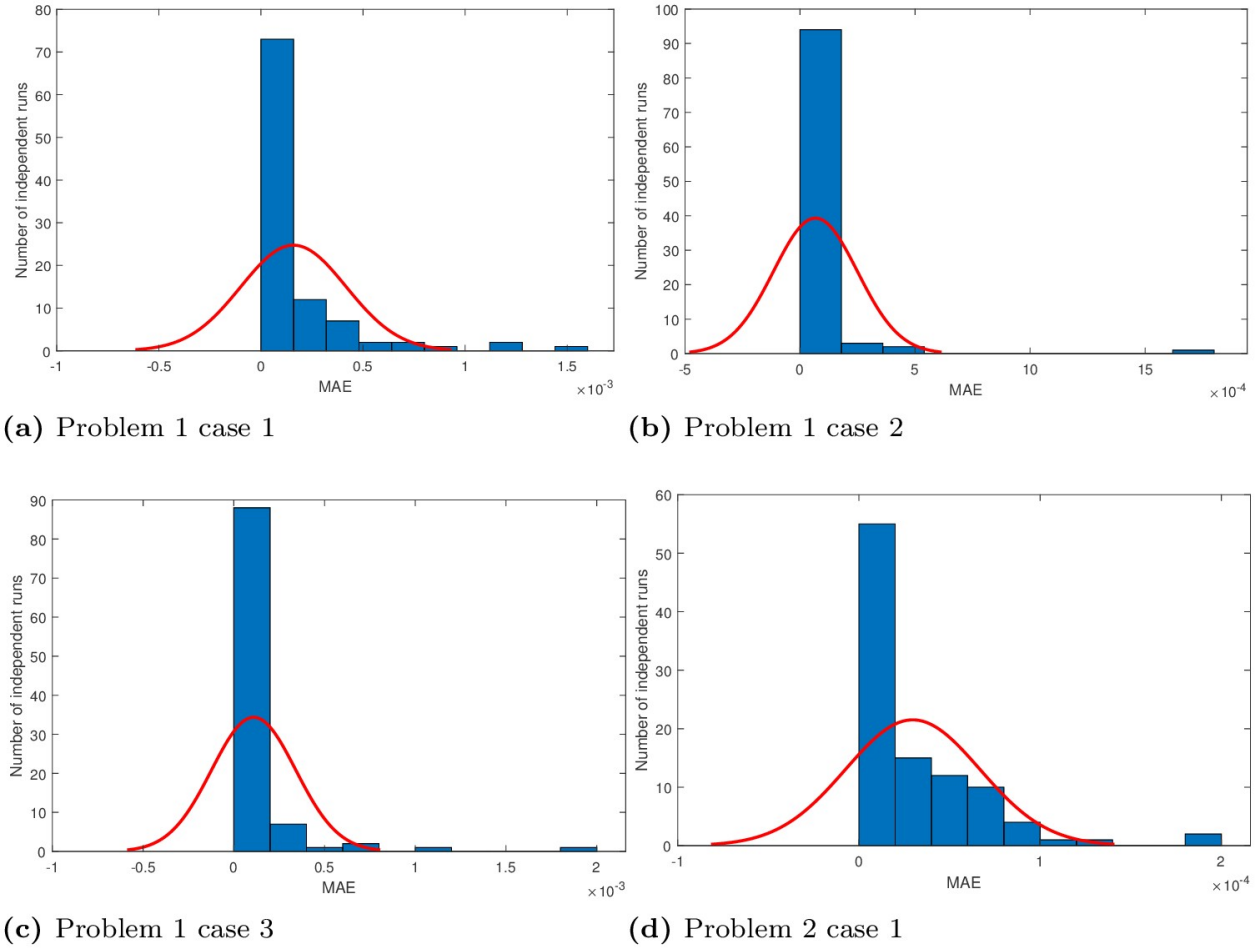
Normal plots of MAE obtained by FO-DPSO during 100 runs.

**Fig 14:**
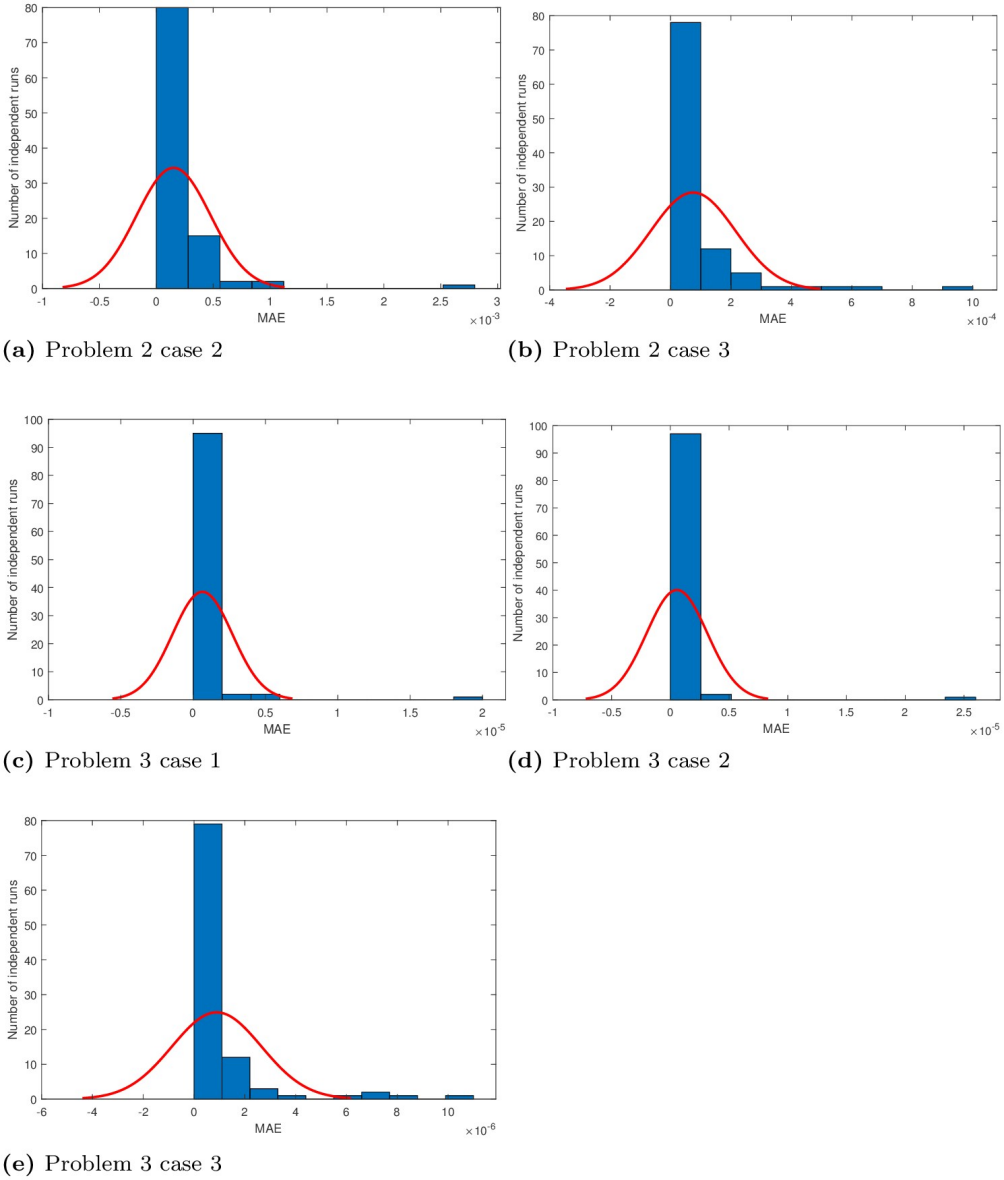
Normal plots of MAE obtained by FO-DPSO during 100 runs.

To verify the stability and robustness of the proposed technique, we got better values of global performance indicators; global mean absolute error (*G*_*MAE*_) as in Eq (50) and mean of fitness values denoted as (*M*_*fit*_) as in Eq (51). All results in terms of these global performance indicators, see Tables 8, 9, and 10 revealed the fact that our approach is better than state-of-the-art approaches reported in the literature [33].

$$\begin{array}{c} G_{MAE}=\frac{1}{R}(\sum_{r=1}^{R}\frac{1}{p}(\sum_{i=1}^{p}|y_{i}-{\hat{y}}_{i,r}|)), \end{array}$$ (50)

$$\begin{array}{c} M_{Fit}=\frac{1}{R}\left(\sum_{r=1}^{R}E_{r}\right), \end{array}$$ (51)

$$\begin{array}{c} MeanTime=\frac{1}{R}\left(\sum_{r=1}^{R}T_{r}\right), \end{array}$$ (52)

where the number of inputs and the number of simulations is denoted by *P*, and *R*, respectively, and *y*_*i*_ denote the corresponding exact solutions and ${\hat{y}}_{i,r}$ the corresponding best approximate values obtained with our approach. *E*_*r*_ represents the best objective value achieved during the *r*^*th*^ simulation. In our experiments, input variable varies from 0 to 1 with *h* = 0.05, *and* 0.2 as step size. Thus, the total grid points were 20, and 6. We repeated our simulations for 100 times. Moreover, mean absolute errors in all solutions are negligible, and histograms with normal distribution fit for mean absolute errors (MAEs) in solutions of problems 1, 2, 3 are given in Figs 13 and 14.

## 6 Conclusion

In this paper, we present a new soft computing approach that combines artificial neural networks with a fractional-order particle swarm optimization (FO-DPSO) algorithm. We conclude this research by following key findings.

- The proposed method to exploit ANNs based FO-DPSO accurately solves variants of non-linear, doubly singular ordinary differential equations according to the computational evaluations.
- We calculate absolute errors in our results using the exact reference solutions, and the errors. Experimental results show that our designed scheme is more accurate compared to the state-of-the-art algorithms GA, and its latest variant GA-SQP.
- We have considered three hard problems with nine cases. A real application is also considered. We have analyzed a mathematical model of porous fins, and temperature profiles are studied for this model.
- We have analyzed several actual application problems. The Values of performance indicators, *G*_*MAE*_, and *M*_*fit*_ dictate that our approach gives results with lower errors compared to other algorithms.
- Frequency graphs of 100 experiments in terms of MAD are presented with normal distribution fittings. These graphs have proved that our approach is reliable and stable in terms of success rate.
- The solutions to the problems are in the appendix to help the reader reproduce the results presented in this paper.
- The proposed approach provides a more accurate solution to differential equations with multiple singularities and systems of such equations. Problems arising in thermodynamics, electromagnetic, and nanotechnology can be handled by the proposed method by changing the activation function of the artificial neural network.

## Supporting information

S1 Appendix (PDF)
