# Supplementary material for: Investigation of singular ordinary differential equations by a neuroevolutionary approach
Source: PLoS One. 2020 Jul 9;15(7):e0235829. doi: 10.1371/journal.pone.0235829 (PMC7347205; doi:10.1371/journal.pone.0235829)
Supplement: S1 Appendix — (PDF) [file pone.0235829.s001.pdf]

Appendix. Derived solutions of the proposed methodology are listed here by using 15 decimal places of accuracy in the values of the weights of ANN models. These solutions are listed with same number as in the manuscript for easy reference.

$$
\begin{aligned}
\hat{y}_{P1C1}(x) = & \frac{-0.821861146351228}{1+e^{-(0.615763276489020x+1.57378328693632)}} \\
& + \frac{-2.05296933597307}{1+e^{-(-2.56484497284092x+2.81821417633090)}} \\
& + \frac{10.8844219088927}{1+e^{-(3.41167784838171x+4.46193289706767)}} \\
& + \frac{8.08400563400337}{1+e^{-(2.80205859340667x-5.27850902305270)}} \\
& + \frac{8.64312648354614}{1+e^{-(2.03246362956917x-4.78500551063410)}} \\
& + \frac{-1.26637188452418}{1+e^{-(-1.64031492220738x+10.9999999529475)}} \\
& + \frac{-5.94556913965137}{1+e^{-(-0.674800743312901x+7.19498980556503}} \\
& + \frac{-0.786722683863799}{1+e^{-(-1.24269117578616x+9.26099615621742)}} \\
& + \frac{-1.17745518552337}{1+e^{-(10.9999999992336x+7.15894660437590)}} \\
& + \frac{3.55149905709368}{1+e^{-(0.138098057518925x+0.158134300529501)}}
\end{aligned} \tag{53}
$$

$$
\begin{aligned}
\hat{y}_{P1C2}(x) = & \frac{5.59797482997324}{1+e^{-(2.33557326043859x-3.04914146795924)}} \\
& + \frac{5.48092723218669}{1+e^{-(3.80186362506428x-6.62955222957144)}} \\
& + \frac{-7.000000000000000}{1+e^{(-0.991978331765235x-7.000000000000000)}} \\
& + \frac{-0.231601892627916}{1+e^{(-5.13768562876876x+11.000000000000000)}} \\
& + \frac{-0.0783373248442701}{1+e^{-(11.000000000000000x+2.99824978271395)}} \\
& + \frac{-7.000000000000000}{1+e^{-(-7.000000000000000x-5.56246148793073)}} \\
& + \frac{2.58704270357355}{1+e^{-(6.32587918316304x-10.9998799173949)}} \\
& + \frac{-1.54728913330909}{1+e^{-(-0.954117521377887x+7.72565335173212)}} \\
& + \frac{0.516802024296291}{1+e^{-(4.07700467472750x+1.20961239149227)}} \\
& + \frac{-0.391405614300637}{1+e^{-(4.10938104962186x+2.42615628057582)}}
\end{aligned} \tag{54}
$$

$$\begin{aligned}\hat{y}_{P1C3}(x) = &\frac{5.76701071356495}{1+e^{-(1.92827498010823x+4.06898826497454)}}\\
&+\frac{-1.31775881953858}{1+e^{-(3.42115667157001x-4.60261958629882)}}\\
&+\frac{2.14215634885725}{1+e^{-(2.55421985646235x+3.10753244121034)}}\\
&+\frac{-1.35676492211213}{1+e^{-(-3.59201854892601x+5.93135221416186)}}\\
&+\frac{-0.434020222301313}{1+e^{-(-6.92898321856203x-5.46944776348845)}}\\
&+\frac{-4.07924123767865}{1+e^{-(-2.93582429151718x+3.54794659637628)}}\\
&+\frac{7.30392217878697}{1+e^{-(3.36913138761505x-6.01009126658378)}}\\
&+\frac{-0.333467508709190}{1+e^{-(-3.43757137091757x+2.03306919755353)}}\\
&+\frac{0.661800005303552}{1+e^{-(2.43420106843954x+1.76513129451664)}}\\
&+\frac{-2.83055996821691}{1+e^{-(0.136961272618987x+0.371967539462392)}}\end{aligned} \tag{55}$$

$$\begin{aligned}\hat{y}_{P2C1}(x) = &\frac{-0.998477278944855}{1+e^{-(-1.35093739860510x+0.673274659061055)}}\\
&+\frac{-1.81774146251136}{1+e^{-(-0.562070189644546x-2.59801637029072)}}\\
&+\frac{0.174980959987578}{1+e^{-(0.244660493204256x+3.97734986671292)}}\\
&+\frac{-1.44484365345102}{1+e^{-(-1.26449349797195x+2.58888314786106)}}\\
&+\frac{7.70734306242308}{1+e^{-(1.53852518020195x-2.99459959096863)}}\\
&+\frac{-6.19274880248326}{1+e^{-(-0.307839030098898x-4.72148947377065)}}\\
&+\frac{0.344661801174088}{1+e^{-(-1.68189470846516x+5.44545864252358)}}\\
&+\frac{8.71154188662676}{1+e^{-(2.81495693788062x-7.000000000000000)}}\\
&+\frac{1.38430723935017}{1+e^{-(-0.681156415300541x+2.58736006336475)}}\\
&+\frac{1.00789775255815}{1+e^{-(-1.70434122083631x+11.000000000000000)}}\end{aligned} \tag{56}$$

$$
\begin{aligned}
\hat{y}_{P2C2}(x) = & \frac{2.73419759441120}{1+e^{-(2.88698508100862x-7.000000000000000)}} \\
& + \frac{3.09092992962269}{1+e^{-(0.430752236915130x-3.78694131667556)}} \\
& \frac{0.960857568049568}{1+e^{-(1.53144550544048x-0.383257261519789)}} \\
& + \frac{4.14371899062455}{1+e^{-(1.74524951918902x-2.51659736331938)}} \\
& + \frac{-7}{1+e^{-(3.54655746221333x+6.28857006944210)}} \\
& + \frac{5.24127351172499}{1+e^{-(2.62767264204992x-5.56098416908710)}} \\
& + \frac{4.96684007884597}{1+e^{-(0.0596489513198691x+0.829333109306141)}} \\
& + \frac{4.72523349886097}{1+e^{-(4.00647849908556x+5.99542927804958)}} \\
& + \frac{-0.995330489482871}{1+e^{-(2.14564670062166x+7.37474363269750)}} \\
& + \frac{1.49016602793925}{1+e^{-(-0.261116391730216x-4.28871241058931)}}
\end{aligned} \tag{57}
$$

$$
\begin{aligned}
\hat{y}_{P2C3}(x) = & \frac{7.26401830350953}{1+e^{-(1.76263527733700x-3.21403000359153)}} \\
& + \frac{-2.16297501299936}{1+e^{-(2.21811610849807x+10.9985849323217)}} \\
& + \frac{8.58068149581900}{1+e^{-(0.669764866585994x+2.16201601774004)}} \\
& + \frac{-1.05026415095753}{1+e^{-(2.30465431133701x+2.81784631518963)}} \\
& + \frac{-0.550937713403025}{1+e^{-(-1.89114600070963x+1.36170418412080)}} \\
& + \frac{-1.17660532683615}{1+e^{-(0.291474701669649x-6.79349937311004)}} \\
& + \frac{-1.46168473501838}{1+e^{-(-2.79087693383624x+7.45713094650379)}} \\
& + \frac{2.32561830204418}{1+e^{-(-1.12800843525908x-3.69972569435526)}} \\
& + \frac{0.0610839108134737}{1+e^{-(5.96278441865368x+3.97056368882739)}} \\
& + \frac{-2.03852040019739}{1+e^{-(-3.69178066062540x+7.25224163192689)}}
\end{aligned} \tag{58}
$$

$$
\begin{aligned}
\hat{y}_{P3C1}(x) = &\frac{-1.76900752951065}{1+e^{-(-1.61867804222187x-4.37594948363637)}} \\
&+\frac{0.343278677322757}{1+e^{-(-0.858688502335111x-2.80595730824365)}} \\
&+\frac{-0.0156439738191471}{1+e^{-(1.49590495294490x+2.45835258272659)}} \\
&+\frac{-1.02847998746579}{1+e^{-(0.276744717563209x-0.8000811558767619)}} \\
&+\frac{-0.00228344343034363}{1+e^{-(2.47600766277755x-1.07467854747611)}} \\
&+\frac{-0.212974387925091}{1+e^{-(-0.929231783376723x-2.09500209306639)}} \\
&+\frac{0.0740339540800605}{1+e^{-(0.701569445433241x-3.83826526702163)}} \\
&+\frac{-1.62566520264321}{1+e^{-(0.487104248090693x+1.70353371401279)}} \\
&+\frac{0.704352786265181}{1+e^{-(-1.61465986206984x-2.79527431343407)}} \\
&+\frac{0.647921048368762}{1+e^{-(-0.378360137324098x-0.109120970246717)}}
\end{aligned}
\tag{59}
$$

$$
\begin{aligned}
\hat{y}_{P3C2}(x) = &\frac{-2.83145282493573}{1+e^{-(0.812764387087534x+2.73340360199915)}} \\
&+\frac{1.91930276825630}{1+e^{-(-0.148814194841562x-0.355244374992351)}} \\
&+\frac{-2.66644207667072}{1+e^{-(0.500523917546711x+1.80403076182328)}} \\
&+\frac{2.76896062848844}{1+e^{-(-0.606542242314468x+5.00000000000000)}} \\
&\frac{-0.280987256929704}{1+e^{-(-1.25385713518309x-0.772018939864148)}} \\
&+\frac{2.58760444456849}{1+e^{-(0.279879761279156x+1.92961532584840)}} \\
&\frac{-2.24900081171692}{1+e^{-(0.364688537946380x+3.31423856336534)}} \\
&+\frac{0.149595982659424}{1+e^{-(2.40026518624920x+2.65403672276501)}} \\
&+\frac{-0.363274637406504}{1+e^{-(0.815465786648859x+0.431230682151447)}} \\
&+\frac{0.169331837483592}{1+e^{-(1.08454628883358x+0.373475110586574)}}
\end{aligned}
\tag{60}
$$

$$
\begin{aligned}
\hat{y}_{P3C3}(x) = {} & \frac{-0.273966452035744}{1 + e^{-(1.90067741616522x+2.80375164289632)}} \\
& + \frac{-0.662221381390501}{1 + e^{-(-0.306034355492465x-1.97405468692909)}} \\
& + \frac{-5.000000000000000}{1 + e^{-(0.404056510680016x-1.66543809751881)}} \\
& + \frac{5.000000000000000}{1 + e^{-(1.23579993461234x-5.000000000000000)}} \\
& + \frac{3.10155121856894}{1 + e^{(-0.881871915586013x-5.000000000000000)}} \\
& + \frac{0.0649886896544054}{1 + e^{-(2.03774342089065x-5.000000000000000)}} \\
& + \frac{-1.23647781867100}{1 + e^{-(0.0679745013709322x-2.23688680892361)}} \\
& + \frac{0.00941954743583786}{1 + e^{-(-5.000000000000000x-5.000000000000000)}} \\
& + \frac{-4.83684720694876}{1 + e^{-(0.181327931789205x+5.000000000000000)}} \\
& + \frac{3.24999491737725}{1 + e^{-(0.488714677086681x-4.56245802819921)}}
\end{aligned}
\tag{61}
$$
